# Comparative Evaluation of Intraarticular Facet Joint Injection Versus Medial Branch Block in Patients With Low Back Pain: A Randomised Controlled Study

**DOI:** 10.7759/cureus.49232

**Published:** 2023-11-22

**Authors:** Anshul, Naveen Malhotra, Amit Kumar, Jyotsna, Kanika Rohilla, Neha Sinha

**Affiliations:** 1 Anaesthesiology, Pandit Bhagwat Dayal Sharma Post Graduate Institute of Medical Sciences, Rohtak, IND; 2 Cardiac Anaesthesia and Pain Management, Pandit Bhagwat Dayal Sharma Post Graduate Institute of Medical Sciences, Rohtak, IND

**Keywords:** fluoroscope, medial branch block, intraarticular injection, low back pain, facet joint

## Abstract

Introduction: Lumbar facet joint pain may refer to the back, buttocks, and proximal parts of the lower extremities. Intraarticular facet joint injections, facet joint nerve blocks, or facet joint neurolytic procedures are popular for the management of facet joint pain.

Material and methods: In this prospective, randomised study, 60 patients with a medical evaluation and pain pattern consistent with lumbar facet joint pain were randomly allocated to two groups. Group Ⅰ (n=30) patients were administered fluoroscope-guided lumbar facet joint injection, and group Ⅱ (n=30) patients were administered fluoroscope-guided lumbar facet joint nerve block. The primary objective is to compare the efficacy of both in managing facet joint pain in terms of pain and disability improvement. Secondary objectives were to compare the requirement for repeat injections and parameters related to the block, such as ease of administering the block, and to note the side effects pertaining to either of the blocks.

Results: There was a statistically significant improvement in pain score after injection in both groups (p>0.05). The mean pain score in both groups remained less than two at all time intervals throughout the study period (p>0.05) Excellent patient satisfaction was reported by the majority of the patients at different time intervals in both groups.

Conclusion: Both lumbar facet joint injection and lumbar facet joint nerve block are safe and effective techniques for managing lower back pain patients. Both techniques provide adequate pain relief and disability improvement.

## Introduction

Facet joints, also known as zygapophysial joints, are diarthrodial articulations between the posterior elements of the adjacent vertebrae. Facet joints provide stability and mobility, allowing the spine to bend and twist. These joints have innervation from the medial branches of the dorsal rami of the spinal nerves at the L1-4 levels. Each segmental medial branch of the dorsal ramus supplies at least two facet joints [1-3].

Lumbar facet joint pain may refer to the back, buttocks, and proximal lower extremities [1-3]. Intraarticular facet joint injections, facet joint nerve blocks, or facet joint neurolytic procedures are popular for the management of facet joint pain. Intra-articular facet joint injections have diagnostic as well as therapeutic value. It has the advantages of a simple technique and localised containment of the infiltrate within the joint. However, its effect depends on the adequate diffusion of drugs into the capsule of the joint [2,4]. Intraarticular facet joint and facet joint nerve blocks have been advised to be performed, ideally under fluoroscopic guidance.

There is limited literature comparing the efficacy of lumbar facet joint injection with lumbar facet joint nerve block in patients with low back pain. Therefore, the present study was planned with the primary objective of comparing the efficacy of intraarticular facet joint injection with facet joint nerve block in managing facet joint pain in terms of pain and disability improvement. Secondary objectives were to compare the requirement for repeat injections and parameters related to the block, such as ease of administering the block, and note the side effects pertaining to either of the blocks.

## Materials and methods

The present prospective, single-blind, randomised controlled trial was conducted at the Pain Management Centre of a postgraduate teaching institute. After approval from the institutional ethical committee vides letter no. SURG/DEAN/17/2864 and informed written consent, 78 patients (ASA I-II) aged between 20 and 60 years were assessed for eligibility. The inclusion criteria were: (1) medical evaluation and pain pattern consistent with lumbar facet joint pain; (2) magnetic resonance imaging (MRI) findings corresponding with the patient’s clinical symptoms; and (3) conservative treatment with oral medication and physical therapy not producing an effective response even after six weeks. Patients who refused to participate with known contraindications for facet joint injection, a history of adverse reactions to local anaesthetics or steroids, malignancy, infection, pregnancy, or lactating females, and a previous history of facet joint injection and spine surgery were excluded. After eliciting a medical evaluation, a detailed review of the imaging studies, including MRI, was done. The numerical rating scale (NRS, 0-10) for pain was explained to each patient in their own language before performing the procedure.

Randomization and Blinding

Sixty-eight patients were randomly allocated to one of the two groups using a computer-generated sequence of random numbers table using coded, sealed, opaque envelopes for allocation. Patients were explained about the block; however, they were blinded to group allocation; hence, the approach of the block was not disclosed to the patients. Group Ⅰ (n=34) patients were administered fluoroscope-guided lumbar facet joint injection, and group Ⅱ (n=34) patients were administered fluoroscope-guided lumbar facet joint nerve block.

Technique for block

Each patient was placed in the prone position with a pillow under the pelvis to attenuate lumbar lordosis. After ensuring full aseptic precautions, cleaning, and draping the area, Lignocaine 1% was infiltrated subcutaneously.

Lumbar Facet Joint Block

**Figure 1:**
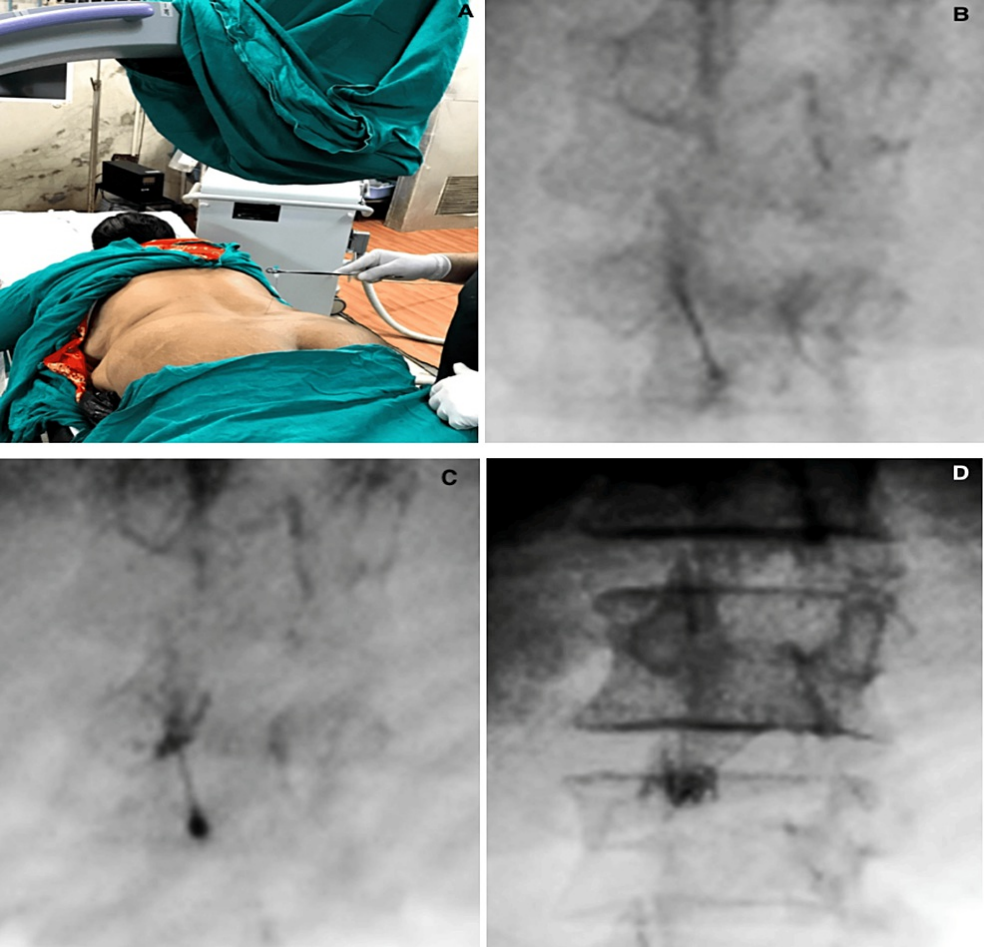
Fluoroscope-guided lumbar facet joint injection. (A) Localization of facet joint; (B) needle advancement in facet joint under fluoroscope guidance; (C) confirmation of adequate needle positioning by injecting contrast medium; (D) confirmation of adequate drug spread under fluoroscope guidance.

A 23-gauge, 3½-inch spinal needle was advanced into the facet joint under fluoroscopic guidance. Adequate needle positioning was confirmed by injecting 0.25 ml of contrast medium (iohexol 350). After the correct placement of the needle, a 2 ml drug solution comprising 0.25% bupivacaine plus 10 mg of triamcinolone was injected into each facet joint (Figure 1).

Lumbar Facet Joint Nerve Block

**Figure 2:**
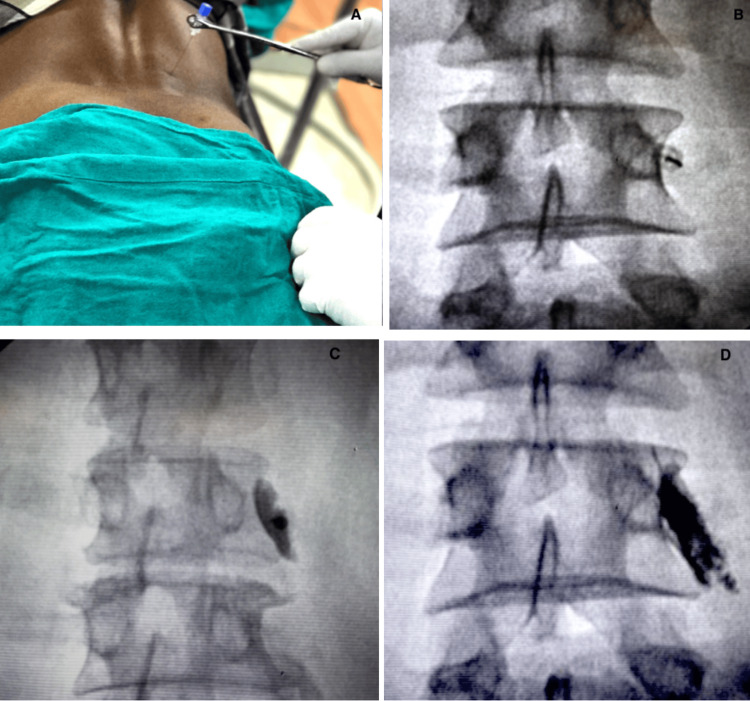
Fluoroscope-guided lumbar facet nerve block. (A) Localization of the dorsal ramus medial branch of the relevant lumbar facet joints; (B) needle advancement under fluoroscope guidance; (C) confirmation of adequate needle positioning by injecting contrast medium; (D) confirmation of adequate drug spread under fluoroscope guidance.

A 23-gauge, 3½-inch spinal needle was advanced at the site of the dorsal ramus medial branch of the relevant lumbar facet joints under fluoroscopic guidance. The adequate position of the needle was ensured by injecting 0.5 ml of contrast medium (iohexol 350). After correct needle placement, a 2 ml drug solution comprising 0.25% bupivacaine plus 10 mg of triamcinolone was administered. For each affected lumbar facet joint, two facet joint nerve blocks were done - first at the affected level and second at the immediate higher level (Figure 2).

Patients were monitored in the recovery area for one hour. Pain was assessed using NRS one hour before the injection, one hour after the injection, and two weeks, one month, two months, three months, and six months after the injection. The RMQ [5] (Roland-Morris Questionnaire, a self-administered questionnaire derived from the Sickness Impact Profile) and ODI (Oswestry Low Back Pain Disability Questionnaire) [6] were calculated one hour before the injection and two weeks, one month, two months, three months, and six months after the injection. Patient satisfaction was assessed two weeks, one month, two months, three months, and six months after the injection on a four-point scale (Excellent: when the pain was completely resolved or diminished by 75% or more; Good: when pain diminished by 50% to 74%; Fair: when pain diminution was 25% to 49%; and Poor: when pain diminution was less than 25% or there was an exaggeration in pain). Patients were followed up for six months after the first injection. Repeat injections with the same previously used approach were administered in cases of inadequate pain relief (NRS > 4).

A maximum of six facet nerve blocks were done in one sitting. No more than three intra-articular injections of each facet joint or lumbar facet joint nerve block were performed during the six-month study period. Any side effects and complications, like pain during administration of the drug solution, pain at the injection site, and swelling, were recorded. Pain during drug administration was assessed on a four-point scale: 1 - no pain, 2 - mild pain, 3 - moderate pain, and 4 - severe pain. The surgery requirement for the presenting problem was assessed, and the number of patients requiring surgery at the end of the six-month study period was recorded.

Sample size estimation

A sample size of 30 per group was calculated to achieve a power of 85% to show a difference of 50% in NRS and 40% in ODI with a type I error rate of 5%. A 40% change in ODI and a 50% change in NRS were found to be clinically relevant in previous studies [4] and were also used for sample size calculation in the present study.

The formula for calculating sample size is given below:

*n* = ($\sigma{_{1}}^{2}$^ ^+ $\sigma{_{2}}^{2}$) . [*Z* _1- α/2_ + (*Z *FLOUROSCP_1-β_)]^2^ (*M*_1 _- *M*_2_)^2^

where *Z*_*α*/2_ is the critical value of the normal distribution at *α*/2 (e.g., for a confidence level of 95%, *α* is 0.05 and the critical value is 1.96), *Z_β_* is the critical value of the normal distribution at *β* (e.g., for a power of 90%, *β* is 0.1), and *M*_1_ and *M*_2_ are the expected sample means of the two techniques. *σ*_1_ and *σ*_2_ are the expected sample SDs of the two techniques.

Statistical analysis

The Statistical Package for the Social Sciences (SPSS) version 17.0 (IBM SPSS Statistics, Inc., Chicago, Illinois, USA) was used in this study for statistical analysis. Differences in age and weight in the two groups were compared with Friedman’s one-way analysis of variance (ANOVA). The change in pain score (NRS 0-10) and change in the ODI score within the two groups at different time intervals were compared by a paired t-test. The Kruskal-Wallis test was used for comparison of the NRS score and the ODI score at different time intervals in the two groups. The chi-square was used to compare gender distribution, patient satisfaction, number of injections, and pain during administration of the injectate amongst the two groups. The results were considered statistically significant if the p-value was ≤0.05.

## Results

**Figure 3:**
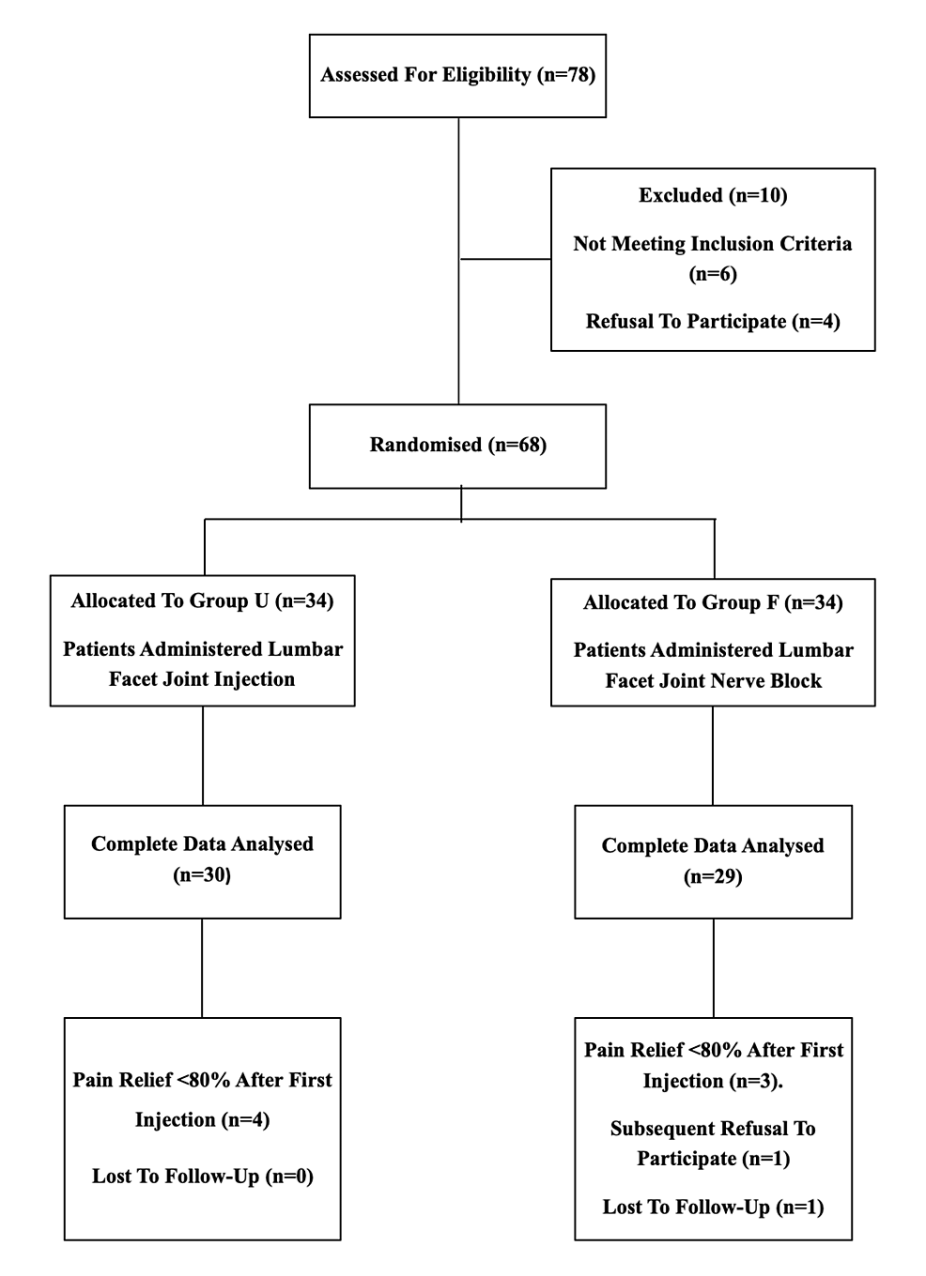
Consort diagram.

A consort diagram has been shown in Figure 3.

The groups were comparable with respect to age, weight, and sex distribution. The mean age of group Ⅰ was 46.93 ± 12.37 and group Ⅱ was 46.83 ± 9.91 years (p=0.971). There were 6 males and 24 females in group Ⅰ, while group Ⅱ consisted of 10 males and 19 females (p=0.211). The mean weight in group Ⅰ was 60.37 ± 7.96 kg and group Ⅱ was 61.62 ± 8.88 kg (p=0.57).

Approximately 80% of patients in both groups underwent block at the L4-L5 and L5-S1 levels. In group I, unilateral block was given in 17 patients as compared to 19 patients in group II (p>0.05), and bilateral block was given in 14 and 11 patients in groups I and II, respectively (p>0.05). Twenty patients in group I and 28 patients in group II had single-level block (p>0.05), and 11 patients in group I required multiple levels of block as compared to only 2 patients in group II (p<0.05).

Table 1 shows the pain score, ODI score, and RMQ score at different time intervals in the two groups.

**Table 1:** Pain score (NRS), ODI score, and RMQ score in the two groups at different time intervals. NRS: numeric rating scale; Inj: injection; p-value*: p-value Mann-Whitney U; ODI: Oswestry Disability Index; RMQ: Roland-Morris Questionnaire.

	Groups	Before Inj	1 hour after Inj	2 weeks after Inj	1 month after Inj	2 months after Inj	3 months after Inj	6 months after Inj	p-value (Friedman’s ANOVA)
Pain score (NRS) at different time intervals (mean ± SD)	Group I n=30	7.63 ± 0.81	1.83 ± 0.91	1.74 ± 0.86	1.43 ± 0.82	1.47 ± 1.2	1.83 ± 1.14	1.8 ± 1.15	<0.001
	Group II n=29	7.72 ± 0.7	1.75 ± 0.59	1.93 ± 0.83	1.89 ± 1.09	1.81 ± 0.96	2.11 ± 1.34	1.9 ± 1.21	<0.001
	p-value*	0.844	0.572	0.524	0.109	0.145	0.12	0.28	
ODI scores (%) at different time intervals (mean ± SD)	Group I n=30	57.29 ± 10.45	-	25.28 ± 8.36	22.63 ± 6.73	16.98 ± 8.60	16.50 ± 9.54	15.08 ± 9.95	<0.001
	Group II n=29	54.80± 9.16	-	27.13 ± 8.88	25.87 ± 10.53	21.52 ± 10.02	19.59 ± 9.18	18.82 ± 8.39	<0.001
	p-value*	0.339	-	0.416	0.071	0.54	0.598	0.416	
RMQ scores at different time intervals (mean ± SD)	Group I n=30	15.70 ± 3.69	-	6.97 ± 4.16	4.67 ± 3.03	3.17 ± 2.20	2.27 ± 1.44	2.17 ± 0.99	<0.001
	Group II n=29	13.0 ± 2.76	-	5.56 ± 3.37	4.0 ± 2.04	2.74 ± 1.29	2.19 ± 0.68	2.15 ± 0.66	<0.001
	p-value*	0.339	-	0.168	0.340	0.383	0.789	0.935	

The results showed clinically and statistically comparable patient satisfaction between the two groups at all time intervals of the six-month study period (p>0.05). Table 2 displays the variation in pain score and ODI score at different time intervals for both groups.

**Table 2:** Change in pain score (NRS) and ODI score in the two groups at different time intervals. NRS: numeric rating scale, SD: standard deviation, B.I.: before injection, ODI: Oswestry Disability Index.

	Groups	B.I. - 1 hour	B.I. - 2 weeks	B.I. - 1 month	B.I. - 2 months	B.I. - 3 months	B.I. - 6 months
Change in Pain Score (mean ± SD)	Group I (n=30)	5.8 ± 1.06	5.89 ± 1.23	6.2 ± 1.21	6.16 ± 1.51	5.8 ± 1.35	5.83 ± 1.56
	Group II (n=29)	5.97 ± 0.88	5.79 ± 1	5.83 ± 1.38	5.91 ± 1.24	5.61 ± 1.5	5.82 ± 1.4
	p-value (unpaired t test)	>0.05	>0.05	>0.05	>0.05	>0.05	>0.05
Change in ODI Score (%) (mean ± SD)	Group I (n=30)	-	2.01 ± 9.74	34.66 ± 9.0	40.31 ± 9.47	40.79 ± 11.26	42.21 ± 11.77
	Group II (n=29)	-	27.67 ± 11.25	28.93 ± 12.51	33.28 ± 13.07	35.21 ± 13.25	35.98 ± 12.44
	p-value (unpaired t test)	-	>0.05	>0.05	>0.05	>0.05	>0.05

The number of patients requiring a second injection during the six-month study period was one each in two groups. It was statistically comparable between the two groups (p=0.11). No patient required a third injection during the study period. A total of 31 injections were given in group I and 30 in group II (p=0.399). There was a time interval of 50 and 96 days between two consecutive injections in groups I and II, respectively (p=0.542). In group I, 18 patients reported mild pain and 10 patients reported moderate pain, as compared to group II, where mild pain was reported by 18 patients and moderate by 8 patients. Only one patient in both groups reported severe pain (p=0.679).

Five patients each in both groups reported injection site soreness. Increased pain was reported by two patients in group I and one patient in group II. Three patients in each group experienced a technical side effect when the needle became clogged by corticosteroid and/or radiocontrast dye.

## Discussion

In the present study, both groups were comparable with respect to age, weight, and sex distribution. Regular engagement in lifting heavy weights as a part of a daily routine like domestic work, agricultural work, labour activities, etc., could explain the female preponderance in this study.

The most common level of block performed in both groups was L4-L5 and L5-S1 (around 80%). These results were comparable to those of Datta et al. [7]. There was a clinically and statistically significant improvement in pain scores after the procedure in both groups at different time intervals. These results were in concordance with other studies [8-10]. However, Marks et al. observed no improvement in NRS scores between the two groups [11]. The follow-up period in their study was three months, as compared to the six-month follow-up in this study. Both groups were clinically and statistically comparable in terms of change in pain score. Similarly, Manchikanti et al. [8] and Shih et al. [10] observed that all patients experienced significant pain relief in both groups for three months, whereas this was variable after three months.

Manchikanti et al. [12] found that a minimum clinically important difference (MCID) of at least 10 to 12 is clinically meaningful for change in ODI. Here, we observed a major improvement in ODI, suggesting that both techniques of lumbar facet injection were associated with overall clinical improvement in physical disability. Similar reductions in ODI scores after lumbar facet injections were reported by other authors [8-10].

In this study, the mean RMQ score was less than 10 after two weeks of the procedure and less than five after one month of the procedure in each group. When the RMQ score at different study time intervals was compared in both groups, it was clinically and statistically comparable (p>0.05). Similarly, greater reductions in the RMQ score were reported by other authors [8-10].

Both groups were comparable in terms of patient satisfaction at all time intervals. The majority of patients reported excellent satisfaction at different time intervals in both groups (p>0.05). Similar results have been observed in other studies [8-10].

We planned to repeat injections if the NRS score was >4. Only one patient in each group required a second injection, and no patient had an NRS score of >4 after the second injection. The total number of injections was comparable between the two groups. In comparison to other studies, we observed better results [9-10,13].

Lumbar facet corticosteroid injections have been advised to be performed ideally under fluoroscopic guidance, which is considered the gold standard for accurate drug placement [2-4]. In our study, we used real-time fluoroscopy during contrast injection, which increased therapeutic value and avoided possible complications.

Although the ease of injection administration between the two groups was not compared in our study, it was clinically easier in facet joint nerve block as compared to facet joint injection. We observed that four patients who had both intraarticular and periarticular spread during facet joint intraarticular injections had good and sustained relief during the six-month study period. Our study results were in concordance with Bani et al. [14].

We did not observe any technique- or injectate-related serious side effects. None of the different types of complications, like infection, reaction to drugs, epidural or subarachnoid blockade, or weight gain, were observed. Patients did not report any muscle spasms, swelling, fever, rash, itching, or other side effects after the injection. A similar side effect profile has been observed in other studies [11,15]. The incidence of adverse events has been reported to be more common when a trainee is involved with the procedure [16]. However, in our study, a single experienced interventional pain specialist performed all injections, which could have led to a lower incidence of side effects.

The present study has several limitations. First, blocks were performed by a single practitioner, which may have limited the generalizability of the study findings. Second, we followed patients for six months only, but they could be followed up to one year to focus on long-term outcomes. Third, it is very difficult to conduct a double-blinded, controlled study with non-traditional modalities such as fluoroscopy. Fourth, our study was not powered to assess the safety aspects and complication rates of the two techniques. A larger patient cohort has to be enrolled to compare adverse effects, and this is not a placebo-controlled study.

## Conclusions

Both lumbar facet joint injection and lumbar facet joint nerve block are safe and effective techniques for managing patients suffering from low back pain. Both techniques provide adequate pain relief and disability improvement. Instead of performing therapeutic facet joint injections with local anaesthetics and corticosteroids after a positive diagnostic injection with local anaesthetics, it is suggested that therapeutic facet joint injections with local anaesthetics and corticosteroids should be done in a single sitting.
